# Benefits of the Use of Lactic Acid Bacteria Starter in Green Cracked Cypriot Table Olives Fermentation

**DOI:** 10.3390/foods9010017

**Published:** 2019-12-23

**Authors:** Dimitrios A. Anagnostopoulos, Vlasios Goulas, Eleni Xenofontos, Christos Vouras, Nikolaos Nikoloudakis, Dimitrios Tsaltas

**Affiliations:** Department of Agricultural Sciences, Biotechnology and Food Science, Cyprus University of Technology, Limassol 3036, Cyprus; da.anagnostopoulos@edu.cut.ac.cy (D.A.A.); vlasios.goulas@cut.ac.cy (V.G.); eleni_xenofontos@hotmail.com (E.X.); ChrisVour@hotmail.com (C.V.); n.nikoloudakis@cut.ac.cy (N.N.)

**Keywords:** fermentation, table olives, microbiological changes, organoleptic, physicochemical

## Abstract

Table olives are one of the most established Mediterranean vegetables, having an exponential increase consumption year by year. In the natural-style processing, olives are produced by spontaneous fermentation, without any chemical debittering. This natural fermentation process remains empirical and variable since it is strongly influenced by physicochemical parameters and microorganism presence in olive drupes. In the present work, Cypriot green cracked table olives were processed directly in brine (natural olives), using three distinct methods: spontaneous fermentation, inoculation with lactic acid bacteria at a 7% or a 10% NaCl concentration. Sensory, physicochemical, and microbiological alterations were monitored at intervals, and major differences were detected across treatments. Results indicated that the predominant microorganisms in the inoculated treatments were lactic acid bacteria, while yeasts predominated in control. As a consequence, starter culture contributed to a crucial effect on olives fermentation, leading to faster acidification and lower pH. This was attributed to a successful lactic acid fermentation, contrasting the acetic and alcoholic fermentation observed in control. Furthermore, it was established that inhibition of enterobacteria growth was achieved in a shorter period and at a significantly lower salt concentration, compared to the spontaneous fermentation. Even though no significant variances were detected in terms of the total phenolic content and antioxidant capacity, the degradation of oleuropein was achieved faster in inoculated treatments, thus, producing higher levels of hydroxytyrosol. Notably, the reduction of salt concentration, in combination with the use of starter, accented novel organoleptic characteristics in the final product, as confirmed from a sensory panel; hence, it becomes obvious that the production of Cypriot table olives at reduced NaCl levels is feasible.

## 1. Introduction

Table olives are an essential element, which is closely related to Mediterranean history. Nowadays, they are considered as the most important vegetables worldwide, with a gross production exceeding 2.7 million tonnes/year [1]. The main purpose of table olive fermentation is to achieve a preservation effect and, in parallel, enhancing the organoleptic attributes of the processed product, hence, meeting consumer’s needs. However, in order to standardize this process and consequently secure the quality of the final product, the study of microbiological and physicochemical descriptors for monitoring the fermentation is a pre-request [2]. Three styles (Spanish, Greek, and Californian) are the most well-known and established commercial types globally [3].

Natural fermentation is mainly driven by yeasts and lactic acid bacteria (LAB), present on olive drupes [4,5]. It has been noted that the LAB is responsible for the fermentation of treated olives (Spanish style). However, in a natural process, LAB and yeasts compete, and in some cases, yeasts can exclusively direct fermentation [6]. Except from these two dominant microorganisms, diverse microbial populations are also participating during olive fermentation, such as several species of *Enterobacteriaceae*, *Clostridium*, *Pseudomonas*, *Staphylococcus*, and molds [7]. These microorganisms via their metabolic activities contribute to crucial aspects, such as organoleptic characteristics (color, texture, flavor, etc.) and safe consumption [8]. In general, LAB activity results in brine acidification, via the production of lactic and other acids, using the fermentable substrates, resulting in pH decrease, providing microbiological control to the final product, hence, extending its shelf life [9,10]. Oppositely, yeasts conduce to the flavor and aroma formation via the production of volatile and other desirable compounds, while, at the same time, they enhance LAB growth and the degradation of phenolic and secosteroid compounds, such as oleuropein [11]. However, the microbiota formation also heavily depends on olive cultivar type since different fruit dimension and composition can affect the microbial dynamics responsible for olive fermentation and sway the sensorial attitudes of the product [12].

During fermentation, major physicochemical changes are taking place. Water-soluble compounds are diffused from olives to the brine, while salt follows the opposite direction, until equilibrium at the end of the brining process [13]. Fermentable sugars are the main source of carbon for microorganisms, providing organic acids, which are essential for the stability and succession of the fermentation process.

Although the physicochemical maturation of olives and brines, during processing, has been thoroughly investigated [2,14,15,16,17,18,19,20], there is no information about Cypriot green naturally fermented olives. Furthermore, significant organoleptic parameters, such as texture and color, are understudied. Both are the main attributes that most affect the consumer’s acceptance and may be strongly affected during processing [19,20].

During olive fermentation, a significant amount of salt is added as a preservative in order to prevent undesirable growth of pathogens and improve the organoleptic characteristics of the final product [18]. However, according to the World Health Organization [21], the daily proposed sodium intake has been set at 5 g. Therefore, one of the main goals of the food industry is to harmonize the global nutritional policies according to this guideline. However, the potential NaCl replacement depends on a plethora of factors, linked to cultivar type, drupe composition, as well as the processing and technological parameters [19,22]. All these parameters should be well inquired before implementation at the industrial scale. Furthermore, the final product must be safe from the microbiological point of view.

Several studies reported microbiological and chemical changes in table olives during spontaneous or controlled fermentation employing different cultivars [2,9,14,23,24]; however, the ‘fermentation map’ of Cypriot green cracked olives have not been charted yet. For the above-cited reasons, the aims of this work were (a) to study the microbial and physicochemical changes of Cypriot green cracked table olives during fermentation process at industrial scale, (b) to identify potential markers associated with the fermentation progress, (c) to accelerate the fermentative process by adding a starter culture, and (d) to study the effect of reducing NaCl concentration in combination with starter culture, in order to produce a secure, nutritious, and healthy final product.

## 2. Materials and Methods

### 2.1. Olives Samples and Fermentation Procedure

Olive fruits (*Olea europaea*) were harvested from a commercial orchard (Novel Agro, Nicosia). All fruits were harvested at the green stage of ripening, based on size uniformity criteria and even external color. After the elimination of the defective fruits, drupes were thoroughly rinsed with tap water to eliminate contaminants.

Subsequently, the olives were cracked and subjected to three different types of fermentation, in duplicate (Biological replicate). A particular amount of olive fruits (20 kg) were placed in plastic tanks of 25 kg capacity filled with brines supplemented with 0.33% *w*/*v* citric acid. The citric acid was added in accordance with the Cypriot industrial standard production procedure of table olives. The process was monitored for 365 days (23 ± 2 °C). The three types of treatments were: (i) spontaneous fermentation in 10% *w*/*v* NaCl, (Control, Olive 7 [OL7], and Brine 7 [AL7]), (ii) and (iii) fermentation inoculated with a starter culture of *Lactobacillus plantarum* (Vege-Start 60’, Chr. Hansen A/S, Copenhagen, Denmark) in (ii) 10% *w*/*v* NaCl (Olive 8 [OL8] and Brine 8 [AL8]) and (iii) 7% NaCl *w*/*v* (Olive 9 [OL9] and Brine 9 [AL9]). The amount of NaCl content to 7% was selected because the aim of the Cypriot olives industry is to reduce the sodium content close to 7%.

### 2.2. Microbiological Analysis

Samples were analyzed at regular time intervals (Days 0, 8, 15, 22, 29, 45, 60, 90, 120, 150, 210, 281, 365) throughout fermentation. They were determined for the total viable count (TVC), *Enterobacteriaceae*, LAB, yeasts, *Coliforms*, *Staphylococci*, using the standard pour and spread plate methods after serial dilutions in 0.85% *w*/*v* saline water (Table 1). In the case of olives, before serial dilutions, 10 g were aseptically transferred to stomacher bags filled with 90 mL of saline solution (0.85% *w*/*v* NaCl) and homogenized for 2 min using a Stomacher at 220 rpm speed (Bug Mixer, Interscience, Saint Nom, France). Volumes of 0.1 mL or 1 mL (spread and pour plate, respectively) of serial dilutions in saline solution were placed in Petri dishes for enumeration of the microorganisms. All samples were analyzed in triplicates.

### 2.3. Physicochemical Analysis

Titratable acidity (TA) was determined by potentiometric titration with 0.1 mol L^−1^ NaOH up to pH 8.3, and results were expressed as a percentage of lactic acid (*w*/*v*). pH was calculated using a pH meter (Hanna Instruments, Luton, UK). The salinity of brines was determined using a salinometer. Electrical conductivity was calculated using a conductivity meter (Mettler Toledo, Zürich, Switzerland). Finally, water potential was determined using a WP4C dewpoint potentiometer, following the manufacturer’s instructions. All measurements were performed in triplicates.

Sugars (glucose and fructose), organic acids (lactic, succinic, tartaric, acetic, citric, and malic), and alcohol (ethanol, glycerol) levels were determined during the fermentation, as described in previous studies [9,16], with some modifications. In 1 mL of brine, 100 μL of HClO_4_ was added, and the samples remained at 4 °C for 24 h, following by centrifugation at 12,000 rpm for 60 min at 4 °C. Then, the supernatants were stored at −20 °C for further analysis. Just prior to the analysis, samples were filtered (using 0.22 μm pore diameter filters). Chromatographic analysis conditions were applied as follows: Column: Aminex HPX-87H, 4.6 mm × 250 mm × 3.5 μm (Bio-Rad, Hercules, CA, USA), solvent: 4.5 mM H_2_SO_4_ in H_2_O, isocratic flow rate: 0.5 mL min^−1^, assay temperature: 65 °C; detectors: refractive index detector for sugars and alcohols, and fluorescence at 210 nm for organic acids, injection sample volume in HPLC: 20 μL. Quantitation (mM) was performed by standard curves generated by chromatographic analysis of the standard solutions of the respective substances at various concentrations.

Total polyphenols and antioxidant capacity of brines and fruits were quantified, followed by the identification and quantification of the main polyphenols (oleuropein, hydroxytyrosol) by high-pressure liquid chromatography (HPLC) (Waters 1525) analysis at regular time intervals throughout fermentation. The extraction of the phenolic compounds was carried out, as reported by Tataridou and Kotzekidou [25]. The determination of total phenolic components using the Folin–Ciocalteu (F.C) reagent was based on the method described previously [26]. The reaction products were measured spectrophotometrically at 765 nm. The results were expressed as mg/g or mg/mL of gallic acid equivalent (GAE).

The antioxidant activity was determined using the stable radical 2,2-diphenyl-1-picrylhydrazyl DPPH (Sigma-Aldrich, Taufkirchen, Germany), according to a procedure described previously [27]. Trolox equivalent antioxidant capacity (TEAC) was used as standard. The results were expressed as mg/g TEAC fresh weight, using the standard curve of Trolox. The measurements were performed three times.

Chromatographic analysis in the extracts was performed using HPLC (Waters 1525). The solvents (mobile phase) used were: Solvent A: 1% acetic acid HPLC grade, Solvent B: 100% acetonitrile HPLC grade, Solvent C: 100% methanol grade HPLC. Chromatographic analysis conditions were as follows: Column C18, 4.6 mm × 250 mm × 5 μm (Sigma-Aldrich, Taufkirchen, Germany), 0–20 min: 95% solvent A + 5% solvent B, 20–40 min: 75% solvent A + 25% solvent B, 40–50 min: 50% solvent A + 50% solvent B, 50–60 min: 5% solvent A + 95% solvent B, 60 min: 95% solvent A + 5% solvent B. Phenolic compounds (oleuropein and hydroxytyrosol) were estimated at the ultraviolet spectrum (254 and 280 nm) using the respectively standards. Results were expressed as means (mg/g or mg/mL) and standard deviations of three replicates.

### 2.4. Color and Texture Analysis

Color determination of table olives was performed during the whole process using a CR200 Chroma Meter (Konica Minolta, Nicosia, Cyprus). The instrument was set to the standard white color (Y = 93.9, X = 0.313 and y = 0.3209 or L * = 94.11, a * = −0.99 and b * = 0.89). The olive color was assessed by taking at least 10 random measurements from the surface of different olives [17]. The color was expressed as L * (bright, dark-low dark color values), a * (negative values indicate green, while positive values indicate redness), and b * (negative values indicate blue, and positive values indicate yellow). Furthermore, reduction in parameter hue angle (h *) corresponded to change in color from green to yellow. Finally, an increase in C * corresponded to a stronger color.

Texture analysis was monitored in whole fermentation by taking at least 10 random measurements of different olives, using a dynamometer (John Chatillon and Sons, New Gardens, NY, USA) carrying a 9.5 mm (length) and 3.2 mm (diameter) piston with a 2 mm cylindrical probe [20]. The test speed was 1.5 mm/s, and the penetration force was expressed in N.

### 2.5. Sensory Evaluation

Olive samples were evaluated organoleptically after 4 months of fermentation by a thirteen-member taste-certified panel (5 males and 8 females, aged from 20 to 45 years old) according to International Olive Oil Council (Regulation COI/OT/MO No 1/Rev.1). Texture, flavor, saltiness, bitterness, acidity, off flavors, and overall acceptance were assessed. Each of these features was rated as follows:

❖ Texture: 0 = soft, 5 = intermediate, 10 = coherent
❖ Flavor: 0 = absence, 5 = moderate, 10 = strong
❖ Salty: 0 = no, 5 = moderate, 10 = very much
❖ Bitterness: 0 = No, 5 = moderate, 10 = high
❖ Acidity: 0 = no, 5 = moderate, 10 = high
❖ Off flavors: 0 = absence, 5 = moderate, 10 = strong
❖ Overall acceptance: 0 = reject, 5 = moderate, 10 = strongly accept

### 2.6. Isolation of the Predominant Microflora

Representative colonies growing on De Man-Rogosa-Sharpe agar (MRS) (LAB) and Sabouraud (yeasts) agar plates were isolated at different stages of fermentation. The isolates were purified by streaking twice on the same medium after phenotypic observation using a light microscope. Pure bacterial and yeast cells were stored at −80 °C using glycerol (20%) for future use.

Finally, in order to detect the presence of the starter culture, rep-PCR genomic fingerprinting was performed on 17 random strains isolated from MRS agar, from brines AL7, AL8, and AL9 at 120 days of fermentation, using the (GTG)_5_-primer (5′-GTG GTG GTG GTG GTG-3′). DNA from each strain was obtained according to Bautista-Gallego [28] and stored at −80 °C. PCR reaction and amplification conditions were applied following a method previously described [29].

### 2.7. Statistical Analysis

Data were subjected to an analysis of variance (one-way ANOVA), using the SPSS 20 software (StatSoft Inc., Tulsa, OK, USA), in order to identify statistically significant differences of microbiological, physicochemical, and sensory characteristics across fermentation treatments. Differences between means were determined by the statistical LSD test at *p* ≤ 0.05. In order to study the correlations between variables and treatments, principal components analysis (PCA) was performed (SPSS 20). Furthermore, two of Pearson’s correlation matrices (among components and between components-treatments) were calculated, and an optimal Kaiser–Meyer–Olkin (KMO) measure of sampling adequacy was established. A hierarchical clustering analysis (HCA) of the correlation coefficients was depicted using the gplots version 3.0.1 (heatmap.2 command; R Foundation for Statistical Computing, Vienna, Austria). Finally, matrices of the original component data were standardized in order to depict (via a hierarchical clustering analysis heatmap) differences in the content of the relative variables.

## 3. Results and Discussion

### 3.1. Microbiological Analyses

**Figure 1:**
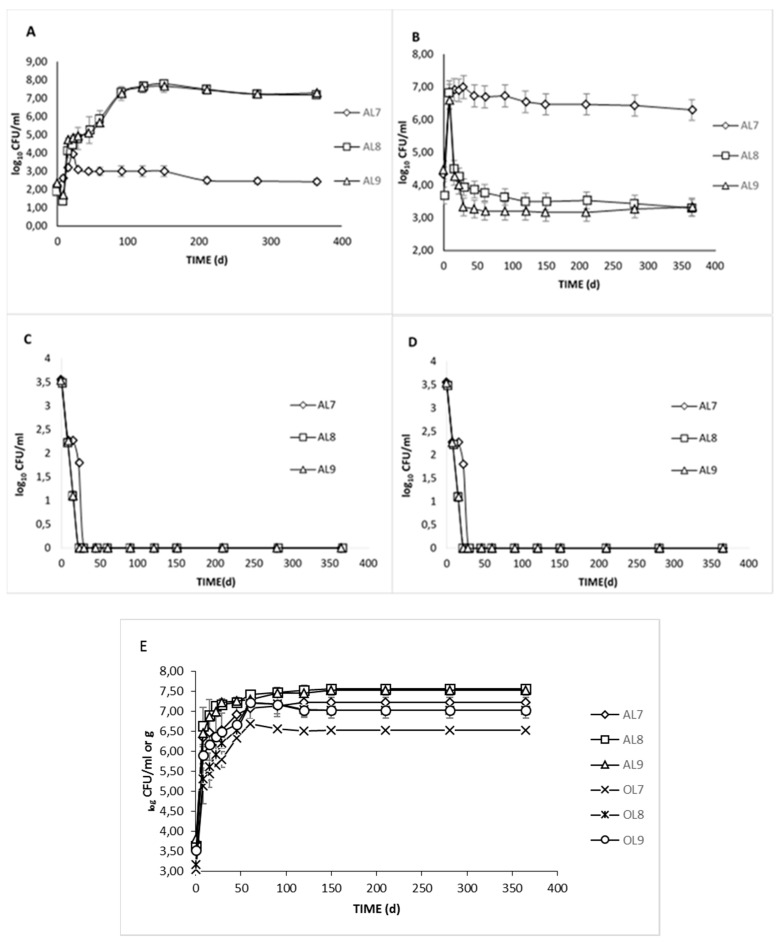
Evolution of microbial changes of spontaneous (◊), inoculated (10% NaCl) (□), and inoculated (7% NaCl) (∆) fermentation of Cypriot green cracked table olives. LAB (**A**), Yeasts (**B**), *Enterobacteriaceae* (**C**), *Coliforms*, and (**D**) TVC (total viable count) (**E**). Data points expressed as log10 CFU/mL of 3 replicates ± standard deviation.

Microbial enumeration was determined in all treatments during fermentation (Figure 1); In general, LAB and yeast numbers were steadily increased and predominated across treatments. On the contrary, *Enterobacteriaceae* and *Coliforms* species were decreased, while *Staphylococci* were not detected during the whole process.

The population size of *Enterobacteriaceae* and *Coliforms* was very similar (no statistical differences were detected) between brines AL8 and AL9 and differed compared to the control during the first days of the fermentation. Specifically, they were detected at an average of 3.5 log cfu/mL at the beginning of the process, but they decreased rapidly and could not be detected after 15 days of fermentation in AL8 and AL9, and after 22 days in AL7, indicating the usefulness of the starter. Indeed, according to Rodriguez-Gomez et al. [30], the use of selected *Lactobacillus pentosus* strains as starters decreased the *Enterobacteriaceae* population faster than in the control treatment. The inoculation contribution to the inactivation of *Enterobacteriaceae* has also been previously noticed [9,17,31]. However, it is obvious that in the present study, *Enterobacteriaceae* decreased more swiftly (about half the time). This could be justified by the use of citric acid at the beginning of the process that led to an early pH decrement at the initial stage of the process, resulting in *Enterobacteriaceae* suppression.

The population of LAB in brine samples changed significantly across the different treatments during fermentation. More specifically, there was an initial increase in LAB counts of AL7 (control) until the 22nd day of fermentation, reaching an average value of 3.95 log cfu/mL. After that peak, a slight decrease was observed, and numbers were retained until the end of the process. The low values of the LAB population in control was in accordance with the literature. LAB populations were limited in spontaneous fermentation, and this was linked to several factors that could have limited the adaptation of LAB in naturally fermented table olives. Some of these factors were the ambient temperature, high salt content, the availability of a source of energy, and natural inhibitory compounds presented in drupes since the fruits were not subjected to lye treatment [23]. On the other hand, in AL8 and AL9, a slight decrease was observed during the first 8 days, followed by a major increase of LAB population, reaching a maximum rate at 120 days (7.67 log cfu/mL and 7.6 log cfu/mL, respectively). No significant differences between these two treatments were observed, except that during the first days of fermentation, LAB populations in AL9 were initially higher. This could be related to the higher diffusion of sugars from olives to the brines due to the reduced NaCl concentration. Moreover, it must be mentioned that the reduction of the LAB population for AL8 and AL9 in the first days of the process indicated an intense competition between the starter with indigenous microflora nutrient assimilation. Indeed, a similar trend was noted in previous studies [17,32], attributed to the lack of nutritional substrates, as well as the presence of inhibitor compounds. According to our data, it was demonstrated that the starter culture withstood the competition with the natural microflora and was not affected by a high salt concentration while predominated in a short period, in contrast to the control treatment. Still, the prevalence of a population during fermentation is a multifactorial process and cannot be always accurately projected. Contrasting to the data of the current study, Rodríguez-Gómez et al. [30] reported that LAB population numbers between inoculated and control treatments had no significant differences. Finally, during the second half of fermentation, a decreasing tendency was noted but always over 7 log cfu/mL, while the population in control was close to the detection limit (2 log cfu/mL).

Yeast growth had an initial lag phase in all cases, occurring across treatments and reaching the maximum level approximately at circa 8 days (7 log cfu/mL), which was in agreement with the literature [2,29,30]. Following, a major decrease in AL8 and AL9 was observed, reaching a value of 3.7 log cfu/mL and 3.5 log cfu/mL at day 60, respectively (Figure 1B). From that point, population levels were maintained steadily until the completion of the process. On the other hand, in AL7, a major increase was observed until the 8th day, and after that, the population reached and retained 7 log cfu/mL level up to the end. As a result, yeasts were the predominant microorganisms in the control treatment. According to the literature, the dominance of LAB in Spanish-style olives has been extremely reported. On the other hand, yeasts are the main organisms driving the fermentation of naturally processing olives [33], although there are studies that have reported the presence of LAB [34]. In the current study, LAB growth in AL7 might have been hampered by salt-tolerant yeast species, resulting in a less acidic product, which was in accordance with the literature [29,35]. Nevertheless, yeast growth is not considered to present any consumption risk during the fermentation of green olives; On the contrary, yeast can metabolize ingredients that enrich the sensory palette and determine the quality of the final product [36]. It is worth noticing that the presence of yeasts, especially in the first days of the process, might have led to the enhancement of the starter culture in inoculated treatments due to their potential production of vitamins and other nutrients, which are mandatory for LAB growth [37].

The microbial composition of TVC in fruits and brines was also depicted (Figure 1E). Overall, the number of microorganisms detected in olive fruits was 1 or 1.5 log lower compared to brines, throughout fermentation. At the early stages, total aerobic counts ranged from 5.2 (OL7) to 5.7 (OL8, OL9) log cfu/g in pulps and from about 6.3 to 6.6 log cfu/mL in brines. The population was increased in all treatments until the 60th day, reaching a maximum value of 7.1, 7.4, 7.3 log cfu/mL for AL7, AL8, and AL9, respectively, while the populations of brined fruits were about 1 log lower than their brines. This magnitude of deviation, among fruits and brine, was also reported in a study carried out on commercialized table olives in Portugal [38]. This finding could be related to the high presence of phenolic compounds in olives, thus, high antimicrobial activity, leading to microbial inhibition, especially in the first 45 days of fermentation.

### 3.2. Physicochemical Analyses

**Figure 2:**
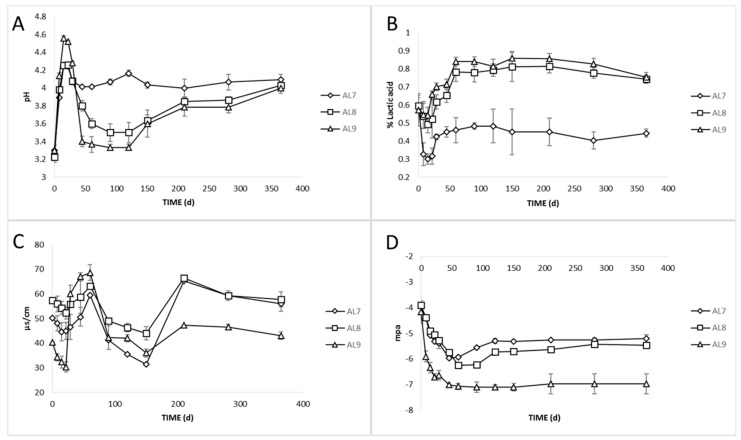
Changes in pH (**A**), titratable acidity (**B**), electrical conductivity (**C**), and water potential (**D**) throughout the fermentation of spontaneous (◊), inoculated (10% NaCl) (□), and inoculated (7% NaCl) (∆) of Cypriot green cracked table olives. Results are expressed as means and standard deviations of three replicates.

The changes in pH in the brines during fermentation of all varieties are presented in Figure 2A. The initial values (Day 0) in all treatments were very low (ca. 3.3) due to the use of citric acid at the beginning of fermentation. After that, there was an increase of about 1–1.5 units until day 22, followed by a major decrease in inoculated treatments (3.5 and 3.3 for AL8 and AL9, respectively). In control, pH remained stable, reaching finally a value of 4 on the 90th day. In all treatments, a slight increase (4, 3.8, and 3.7 for AL7, AL8, and AL9, respectively) was observed, which was stabilized thereafter at about pH 4 at day 365. No differences between treatments were observed at this time point. It was crucial to mention that the fast acidification in the brine matrix was a crucial preliminary step for the succession of fermentation process; pH in brines below 4.5 preserved table olives from spoilage and pathogen microbial growth during fermentation. Furthermore, it had to be noted that the positive effect of the starter in pH drop was profound, especially in the first days, which was in agreement with previous studies [9,18,39].

The reverse change was followed on titratable acidity, as expected (Figure 2B). The highest values were recorded in AL8 and AL9 due to the dominance of LAB (0.81 and 0.86% lactic acid, respectively). It was notable that the effect of initial acidification with citric acid was evident for high values of titratable acidity in the brines during the first days. The titratable acidity was higher in AL9 until the 29th day, and, thereafter, no significant differences were observed between inoculated samples. The higher values in AL9 at the first days of fermentation could be explained due to the low salt concentration, which allowed the faster diffusion of sugars from olives to brines, and thus the faster start of fermentation from LAB. The titratable acidity levels found were in accordance with the LAB enumeration and pH values, described above. Moreover, it is noteworthy that a value of more than 0.48% lactic acid in AL7 was not reached at any time, probably due to the dominance of yeasts, in combination with the weakness of LAB to produce lactic acid due to their low population. In another study [16], fermentation of table olives driven by yeasts attained a final pH close to 4.2–4.3, which was in good agreement with the final pH values of AL7 reported in the present study. However, even though yeasts were the dominant microbial group, the final values for pH and acidity were within the limits of the trade standard applying to table olives of the IOC (2004), where for natural fermentation, the maximum limit for pH and minimum acidity should be 4.3 and 0.3%, respectively. Notably, the higher acidic environment in AL8 and AL9 samples are enough to prevent the growth of spoilage and/or pathogen microorganisms, and thus they may provide an added value to the product. The latter could be confirmed by the faster elimination of such microorganisms, as mentioned above. Thus, our findings suggested that the use of LAB starter culture had a significant effect on the acidification of the brines, achieving a more controllable and successful fermentation.

During fermentation, the production of higher acidity in inoculated treatments caused an increase in electrical conductivity. The pH curve represents the kinetics of the production of H^−^ ions, while that of electrical conductivity represents the production of all ionic species [40]. Figure 2C shows the changes in electrical conductivity during the whole process. As was clearly observed, there was an initial decrease in all treatments until day 22, followed by a major increase until the 60th day. Significant differences were observed in all treatments, while AL9 had the highest values from day 29 to day 60, followed by AL8 and AL7. This was in accordance with pH and acidity scores at this time point, as described above, indicating a clear correlation between the three parameters, confirmed by HCA, as well. In a previous study [41], it was also demonstrated a curvilinear relationship between pH and conductivity during mixed coagulation of milk. However, according to our knowledge, this is the first study indicating the correlation between these parameters in table olives fermentation. Thus, electrical conductivity could be used as a potentially useful tool for table olives monitoring during the fermentation process.

Regarding the water potential of olive fruits, there was a clear difference between OL9 and the other two treatments during the whole process (Figure 2D). The initial values of the three treatments were about −3.9 Mpa. There was a decrease during the first 60 days, where OL9 was statistically higher compared to OL7 and OL8 olive fruits. After that period, the values of all treatments started to have a slightly increasing trend up to the 120th day and then remained stable until the end of the process. Water potential expressed the tendency of the water to move from the fruit to the brine and was related to the expression of osmosis. Thus, it was clear that osmosis pressure in OL9 was higher than in the other two treatments, allowing the faster diffusion of flesh tissue components (sugars, organic acids, polyphenols, etc.) to the brines. Indeed, Papadelli et al. [9] reported that the slow extraction of soluble components from the olives to the brine was related to high NaCl concentration. This is the first report proposing the use of water potential as a tool for soluble component kinetic estimations of table olives during the fermentation process.

Finally, salinity in the brines was monitored throughout fermentation, and adjusted to the initial values of 10% and 7% for each treatment, by periodic dry salt additions in the brines. Salt equilibrium was reached in ca. 3 months and until the end of the process, salt concentration was maintained to its initial values.

**Figure 3:**
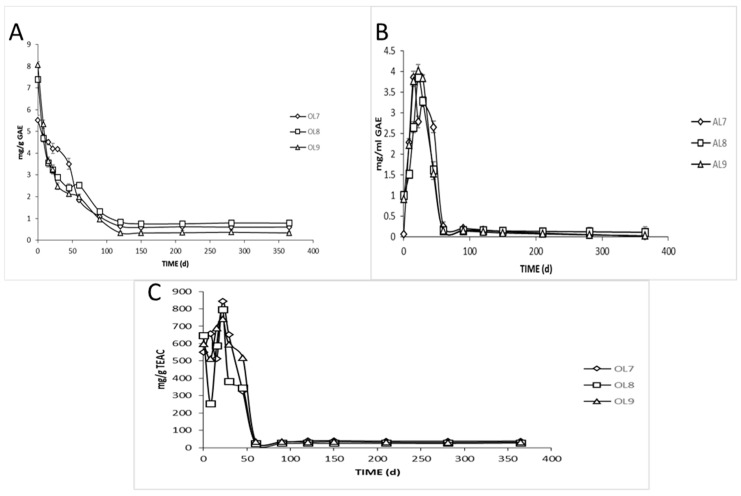
Total phenolic content of olive pulps (**A**) and brines (**B**) and antioxidant capacity of olives (**C**) during spontaneous (◊), inoculated (10% NaCl) (□), and inoculated (7% NaCl) (∆) fermentation of Cypriot green cracked table olives. Results are expressed as means and standard deviations of three replicates, equivalent of mg/g or mL.

The total phenolic evolution of fruits and brine samples is presented in Figure 3A,B, respectively. As clearly observed, the profiles of total phenolic content were quite similar across treatments. Olives exhibited a major loss in total phenolic content during fermentation mainly due to their degradation by LAB and yeasts and secondary due to their diffusion to the brine, as well. A similar trend has been noted by other studies [13,42]. During the first 45 days of fermentation, the decrease rates of phenolic contents were estimated to 37%, 68%, and 75% for OL7, OL8, and OL9, respectively. After 120 days of brining, phenolic content attained a steady-state in traces, with no differences between treatments. The total reduction of phenolic contents was about 88% for all treatments. In fact, the diffusion of phenolic compounds from olive flesh to the brine depended on several parameters, such as cultivar characteristics, fruit skin permeability, type of polyphenols presented in olive flesh, brine concentration, and their ability to diffuse outside the fruit due to accidental or purposely made fruit damage (cracked or razor slitting). As expected, the total phenolic contents in brines increased gradually in all fermenters to rich maximum concentrations of 3.24, 3.85, and 4 g GAE/g after 29, 22, and 22 days of fermentation for AL7, AL8, and AL9, respectively. After the 29th day of brining, the phenolic content started to decrease. This decline might be due to the degradation of phenolic acids by *Lactobacillus plantarum*. It has been demonstrated that *Lactobacillus plantarum* contains phenolic acid decarboxylases, which decarboxylate different phenolic compounds to their corresponding vinyl derivatives [43]. However, it is obviously an analogous reduction of total phenols in control treatment, in which, as previously mentioned, yeasts were the leading microorganisms. This indicates a high enzymatic activity of indigenous yeasts in the degradation of phenolic compounds, which is in accordance with the literature, where it has been reported the important role of yeasts in the olive debittering process [2]. This finding could explain the similarities of total polyphenols loss between inoculated and control samples from the 60th day of fermentation and thereafter.

Additionally, the loss in phenolic compounds resulted in a remarkable loss of antioxidant capacity in olive fruits, as well (Figure 3C). No significant differences between different fermentations were observed after 60 days. The loss of antioxidant capacity transcended to 90% for all treatments.

**Figure 4:**
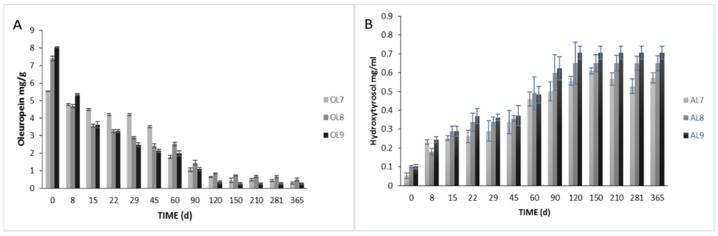
Evolution of oleuropein (**A**) and hydroxytyrosol (**B**) during spontaneous (7), inoculated (10% NaCl) (8), and inoculated (7% NaCl) (9) fermentation of Cypriot green cracked table olives. Results are expressed as means (mg/g or mg/mL) and standard deviations at different times of fermentations.

A major decrease of oleuropein was observed in olive fruits, mainly due to its diffusion to brines and its degradation caused by enzymatic activity (Figure 4A). No significant differences were observed between treatments at the end of the process, which agreed with the trends in total polyphenols values described above. However, the faster-decreasing values of oleuropein in inoculated treatments until the 45th day were notable. At this time point, the reduction reached 62% and 69% for OL8 and OL9, respectively, while, in control, it was no more than 24%. This finding indicated that the inoculated samples were ready to eat in a shorter period. Furthermore, the reduction of oleuropein was also accompanied by an increase in its hydrolysis product in brines (hydroxytyrosol), where the inoculated treatments had significantly higher values after 90 days of fermentation (Figure 4B). This finding confirmed that the enzymatic activity of the starter culture was higher, affecting the secosteroid glucosides and their aglycon derivatives. In line with our findings, in previous studies [43,44], hydroxytyrosol was referred to as the main phenolic compound found in the brines inoculated with the commercial starter. This compound has been mainly linked to the hydrolysis of oleuropein [13] being an important biophenol belonging to the odiphenol group with special antioxidant activity [45], and it has been considered as a marker for the determination of olive debittering [46].

**Figure 5:**
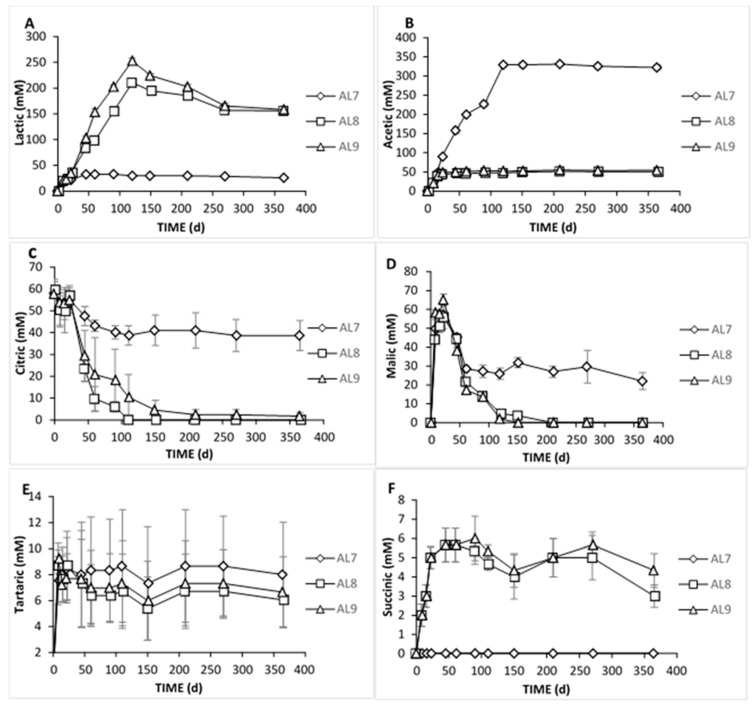
Changes in the concentration (mM) of organic acids (lactic, **A**; acetic, **B**; citric, **C**; malic, **D**; tartaric, **E**; and succinic, **F**) during spontaneous (◊), inoculated (10% NaCl) (□), and inoculated (7% NaCl) (∆) fermentation of Cypriot green cracked table olives. Data points are expressed as means and standard deviations of three replicates.

The changes in the concentration of organic acids in the brines are shown in Figure 5. Significant differences between the three treatments were observed during the whole process, as detected by HPLC analysis. More specific, citric acid was the main acid at the initial stage in all treatments due to its use at the beginning of the fermentation. After 22 days, in AL7, acetic acid became the main acid with considerable presence, while a slight reduction of citric from day 45 to day 120 was observed. However, the concentration of acetic was increasing until day 120 and then remained stable. Its presence in control could be related to yeast metabolism, as well as to the potential of heterofermentative LAB able to produce acetic acid under particular conditions of environmental stress as well as from the metabolism of citric acid [47]. Furthermore, malic and tartaric acids were also found in brines and were increased until day 22, indicating their presence in olive fruits and diffusion to the brines the first days of the process. The latter was in line with the literature, as well [9]. Afterward, these two acids remained unchanged until the end of the process. Thus, there was not any metabolic activity for those acids in AL7. Finally, lactic acid was also detected in the brine AL7 in concentrations not exceeding 32 mM throughout the process, which was related to the low populations of LAB found in the microbial enumeration. The low values found for lactic acid were in accordance with previous studies [16,37]. However, as expected, lactic acid was the main acid in inoculated treatments due to the predominance of LAB. Significant differences between these two treatments were observed from day 45 to day 120, where the concentration of lactic acid in AL9 was higher than in AL8. This was in a combination of pH and titratable acidity values described above. Lactic acid presented a gradual increase until day 120, reaching statistically significant higher values in AL9, followed by a steady decline thereafter. This indicated potential assimilation of lactic acid from yeasts after 120th day due to their high population, which was in accordance with the literature [6]. Citric acid was the main acid at the initial stage due to its use at the beginning of the fermentation. From day 22 to day 60, a major decrease was observed in both treatments (no significant differences), which was related to its microbial degradation to acetic acid [9]. Succinic acid was also determined in the inoculated treatments, the evolution of which was found to be similar to that of the acetic acid, for the same reason, as well (no differences between treatments). Furthermore, regarding malic and tartaric acids, there was an obvious initial increase during the first 22 days, followed by a major decrease and total disappearance of malic acid in about 120 days, while the tartaric acid remained steady until the end. This finding was in agreement with results reported previously [14,24], where malic acid detected in traces at the beginning of the process and decreased at the end of the fermentation period. Moreover, the gradual decrease of malic acid in brines observed during the fermentation of green olives is attributed to its microbial degradation to lactic acid and CO_2_ [9,31]. Finally, for tartaric acid, it has been reported that yeast and other microorganisms are unable to metabolize it [48].

**Figure 6:**
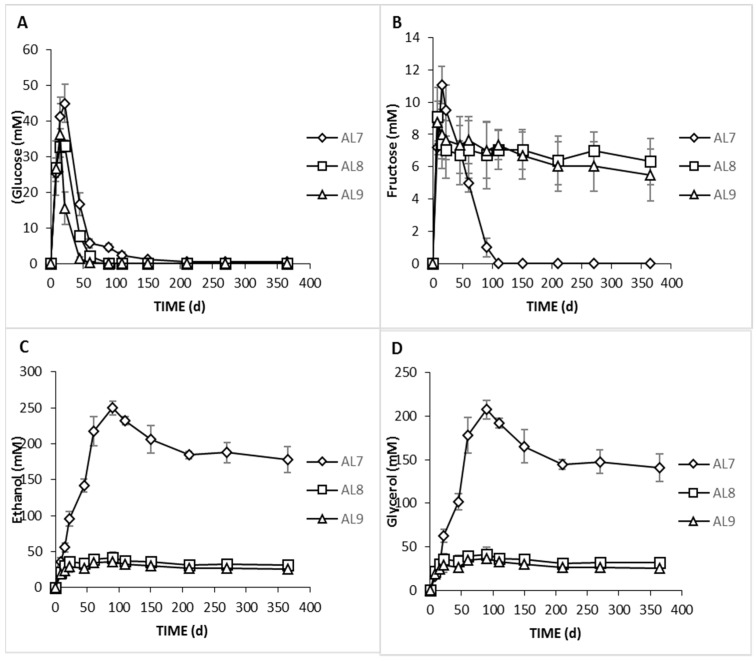
Changes in the concentration (mM) of soluble sugars (glucose, **A**; fructose, **B**) and alcohols (ethanol, **C**; glycerol, **D**) in the brines during processing of Cypriot green cracked table olives of Spontaneous (◊), inoculated (10% NaCl) (□), and inoculated (7% NaCl) (∆) fermentations. Data points are expressed as means and standard deviations in triplicate.

Sugars diffused from fruits into the brine are the main nutritional elements for microbial growth and fermentation. According to our results, glucose and fructose were the main sugars found in the brines as it emerged by HPLC analysis (Figure 6). Glucose was steadily increasing the first days of fermentation, exhibiting the highest value at day 22 for AL9 and day 29 for AL8 and AL7. This could be confirmed by the faster diffusion of the sugar observed from olives to brine in AL9 because of the lower NaCl concentration. A major decrease was recorded thereafter since it was consumed for microbial growth. In fermentation AL9, this decrease was observed earlier (at day 45) compared to fermentation AL8 (day 60) and AL7 (day 90), which was in accordance with previous research, where it was reported that in the inoculated olives, the decrease of glucose was faster than in control [9]. It was notable that at the end of the process, glucose disappeared, but there was a remaining amount of ca. 0.5 mM in the AL7. A similar trend was found for fructose content in AL7. Its amount never exceeded 12 mM, and it was not found after 120 days of fermentation, while it was detectable in the same concentrations (ca. 7 mM) in AL8 and AL9, until the end of the fermentation. The total depletion of fructose in AL7 could be related to some fructophilic yeast species, a fact that agreed with the results of control treatment in the present study [9].

Concerning ethanol, it is mainly related to yeast production activity, having a crucial impact on the sensory properties of naturally fermented olives [29]. Its concentration in AL7 increased gradually until day 90 of fermentation, reaching values 250 mM, followed by a minor decrease thereafter until the end of the process, where it was maintained at ca 178 mM. Similar trends were previously reported [2]. However, ethanol was detected in traces in inoculated treatments because of LAB dominance, confirming that yeast metabolic activity was affected by the inoculation of table olives. Another important product from yeast activity is glycerol [49]. Its presence has been linked with cell protection from osmotic stress [29]. According to our results, it was present in high concentration in control, as expected, while it was very limited in the other treatments (ca. 30 mM during the whole process). In AL7, its concentration increased gradually until the 90th day of fermentation in levels exceeding 207 mM, followed by a decrease afterward, reaching final values of ca. 140 mM. The presence of this compound in naturally fermented table olives has been reported previously [16,37], which was in good line with the present study. It has been noted that the presence of both compounds (ethanol and glycerol) can, in turn, affect crucial organoleptic characteristics, such as texture maintenance and aroma formation [36,50].

### 3.3. Firmness and Color Evolution of Olives

**Figure 7:**
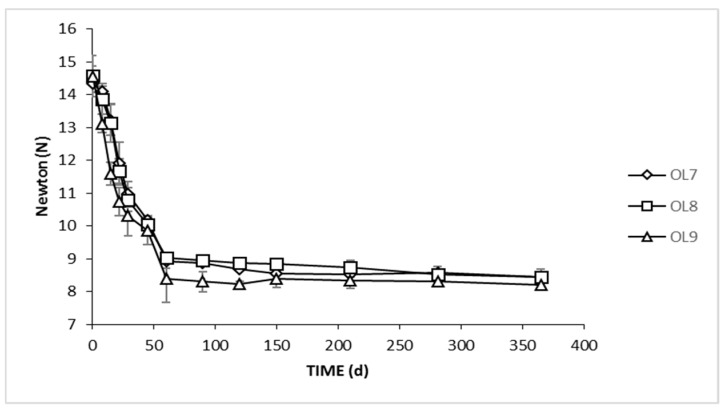
Evolution of texture of olive fruits during spontaneous (◊), inoculated (10% NaCl) (□), and inoculated (7% NaCl) (∆) fermentation of Cypriot green cracked table olives. Data points are expressed as means (N) and standard deviations of 10 random measurements.

The texture has a great impact on consumer’s acceptance of a product, while in main cases, it is considered to be the most important property [51]. The results of textural analysis during the whole process are presented in Figure 7. It could be observed that the values were being decreased as time passed until the 60th day, and after that remained steady until the end of fermentation, in all treatments. The values of OL9 were significant lower until day 29. This could be explained by the lower NaCl concentration. However, no significant differences were observed thereafter, indicating that neither lower NaCl nor brine inoculation affected the texture profile of the final product. Similarly, Fadda et al. [20] investigated the effect of brining time on the texture of naturally fermented green olives, reporting a texture decrease after 30 days of brining. Texture loss is strongly influenced by the enzymatic activity of dominant microflora and, in some cases, may cause softening due to the degradation of pectic substances of the cell wall and middle lamella [52]. The latter depends on crucial brine conditions, such as sodium content and pH [20].

The color attribute of food products is another crucial factor in the acceptance of a food product. The color parameters of olives are listed in Supplementary Table S1. In general, no significant differences were observed between treatments in any of the parameters. Exceptions were the h* and C* parameters. The latter, in the inoculated treatments, were significantly lower than control. This parameter was related to the volume of color, which accounted for a shift to the dark-green zone. Furthermore, there were no significant differences in b* parameter for control and inoculated samples starting from a value of 33 ± 4.4, reaching a decreased value until 45th day (22.6 ± 6), and thereafter remained unchanged until the end. The decreasing values indicated lowering in yellow color. A similar tendency was observed for the parameter L* (no differences), which decreased until day 45 after the fermentation process. The value of the parameter L* was an indicator of the degree of lightness. However, according to the literature [53], light color is associated with a low pH value, which was not in agreement with the present study; otherwise, the inoculated samples should have higher lightness than control. This could be explained by the fact that the lightness parameter is mainly variety dependent. Finally, a major increase in a* parameter was observed in all treatments, demonstrating a distinct toning from green to red, starting for values of about −13 at the beginning of the process, while values of about −1.9 ± 1 were reached at 120 days and thereafter no changes were recorded. This effect could be attributed to the presence of chlorophyllase in the first days of fermentation, leading to hydrolysis of phytol or chemical oxidation reactions [54]. In general, natural green olives had high values for a* parameter, resulting in reddish tones. Finally, the loss of h* was faster in control and lowered significantly until the 150th day, indicating faster brownish coloration. The latter, among other organoleptic characteristics, makes this product less attractive [53], and thus this is another positive effect of inoculated samples.

### 3.4. Sensory Evaluation

**Figure 8:**
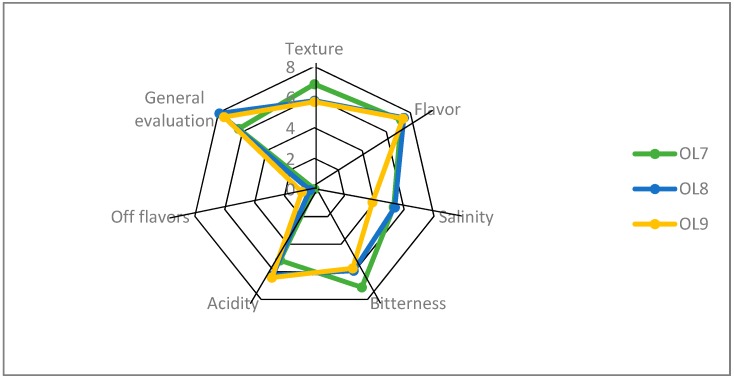
Sensory profiles of spontaneous (OL7), inoculated (10% NaCl) (OL8), and inoculated (7% NaCl) (OL9) fermentation of Cypriot green cracked table olives at 120 days of fermentation.

The organoleptic profile of the samples is presented in Figure 8. In general, the samples were characterized by low remaining bitterness, good texture, and satisfactory acidic taste and odor. No off flavors were noticed in any samples. Overall, differences among treatments were detected on a bitterness descriptor, in which OL7 had a higher score from the other two. The higher contents of both ethanol and glycerol in the control sample was in line with the higher score to the bitterness and lower score of the acid descriptor, according to panelist evaluation. A similar trend was observed for the saltiness score, with a lower value scored in OL9 samples. However, no differences were recorded to a flavor descriptor. Regarding texture, OL8 and OL9 had lower scores. However, they received an equal value of acidity, which was higher than control, while they had the highest score for the overall acceptability descriptor. The most important attribute that influenced the judgment of the panelists was salt content, acidity, and bitterness to a lesser extent, as could be concluded by the scores of those parameters, in combination with the overall acceptability scores.

### 3.5. Multivariate Analysis

**Figure 9:**
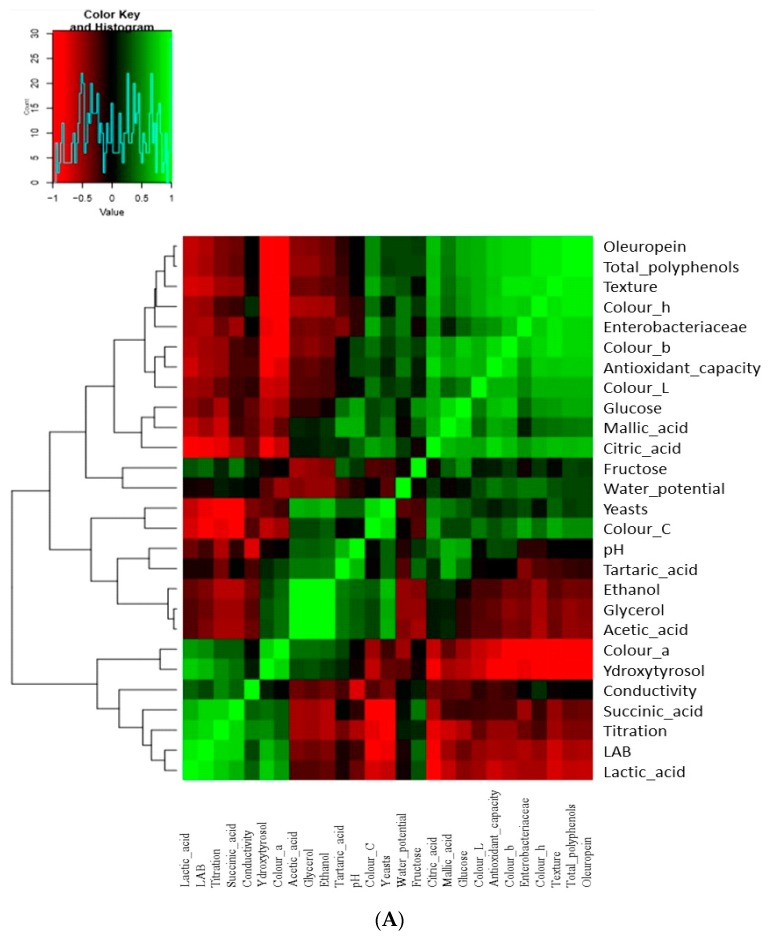

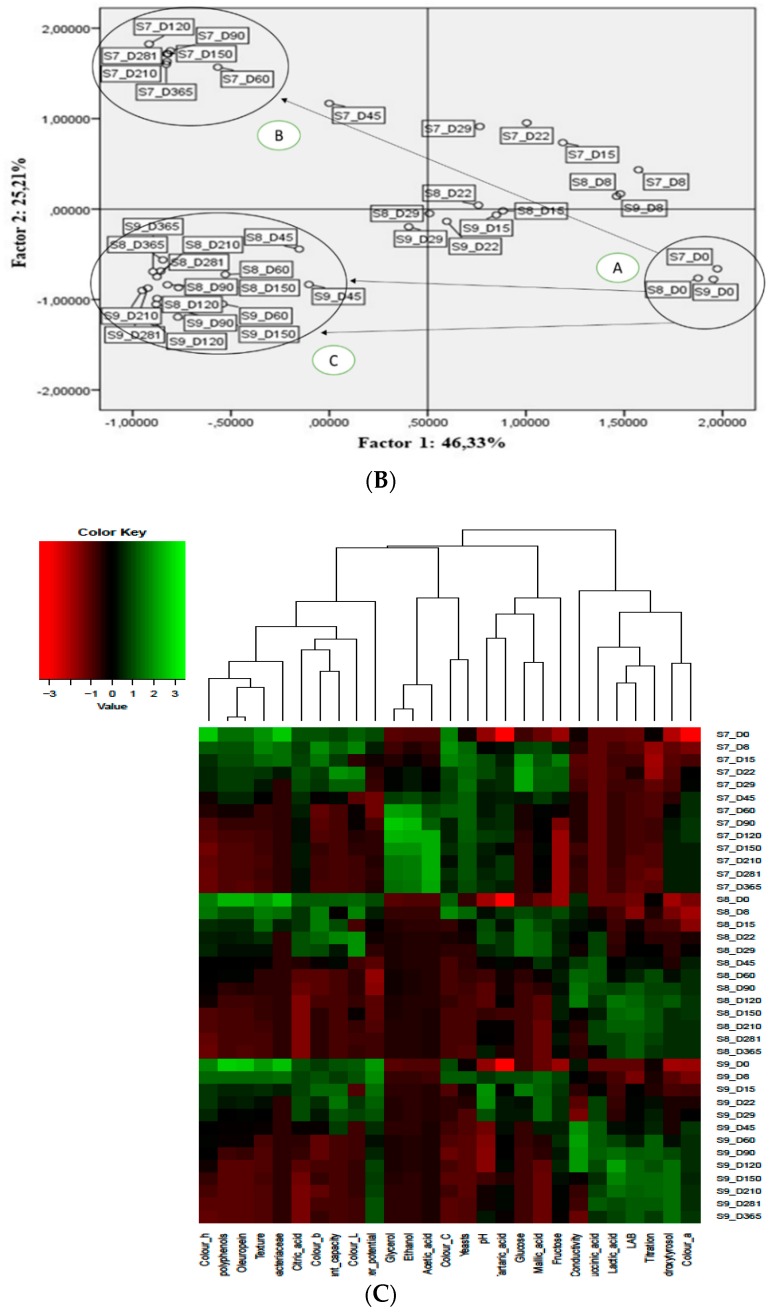
(**A**) PermutMatrixEN analysis between microbial and physicochemical profiles of spontaneous, inoculated (10% NaCl), and inoculated (7% NaCl) fermentation of Cypriot green cracked table olives. (**B**) The plot of scores and loadings between treatments formed by the first two principal components from the PCA (principal component analysis) analysis. Labeling of data points indicates the processing treatment of olives (S9: inoculated and 7% NaCl concentration, S8: inoculated and 10% NaCl concentration, S7: control) and fermentation time (D: Days). (**C**) Heatmap showing the similarities in the observed microbial and physicochemical profiles between the three experiments during fermentation days. Labeling of data points indicates the processing treatment of olives (S9: inoculated and 7% NaCl concentration, S8: inoculated and 10% NaCl concentration, S7: control) and fermentation time (D: Days).

PCA between all studied variables resulted in four eigenvalues greater than 1, explaining an overall 88.84% of the total variance in the dataset, while the first two components explained 71.5% of the distribution (Figure 9B; Supplementary Table S2). PC1 was correlated with LAB, *Enterobacteriaceae*, texture, all color parameters, polyphenols, antioxidant capacity, lactic, citric, succinic acids, and glucose, while PC2 dealt with yeasts, acetic, tartaric acids, ethanol, and glycerol. PC3 was linked with the pH, fructose, malic, and tartaric acids. Finally, PC4 was related to conductivity and water potential. Furthermore, correlations between microbial and physicochemical data are shown in Figure 9A. Among organic acids, lactic and succinic acids were negatively correlated with yeasts and positively correlated with LAB. Oppositely, acetic, tartaric, and malic were positively correlated with yeasts and negatively with LAB. Zooming on the metabolomics, the acetic acid was positively correlated with ethanol and glycerol, confirming the results for control treatment, described above. Oleuropein, antioxidant capacity, total phenols, texture, and color parameters L*, b*, h* were closely related to each other, and all of them were negatively related to LAB and positively related with yeasts. Finally, hydroxytyrosol seemed to be highly correlated with LAB, confirming our results described above.

Regarding correlations between treatments, PCA grouped them into three clusters, clearly characterized based on inoculated treatments versus the control one during fermentation time, as control treatments being separated from inoculated treatments from the 45th day and thereafter (Figure 9B). Inoculation was apparently the most important treatment in sample distribution throughout fermentation. PC1 could be related to fermentation time since a gradual transition of time was noticeable from the right to the left part of the plot. It is crucial to mention that the reduction of NaCl concentration (AL9) did not affect the groups’ distribution. This was a very promising finding, indicating that the NaCl reduction was an achievable goal for the table olives industry.

Furthermore, similarities in the observed microbial and physicochemical profiles between samples are presented in Figure 9C. In detail, in agreement with PCA, inoculated samples had similar profiles to each other after the 45th day, showing a negative correlation with total polyphenols, oleuropein, texture, color h*, color L*, color C*, and malic and citric acid, while they were positively correlated with LAB, titration, lactic and succinic acid, color a*, pH, glucose, and hydroxytyrosol. On the other hand, control treatment was closely related to yeasts, texture, acetic acid, ethanol, and glycerol, while it was negatively related to the positive parameters of inoculated treatments and fructose. Therefore, the multivariate analysis confirmed the different metabolic pathways between non-inoculated and inoculated treatments during fermentation.

### 3.6. Detection of the Presence of the Starter Culture

**Figure 10:**
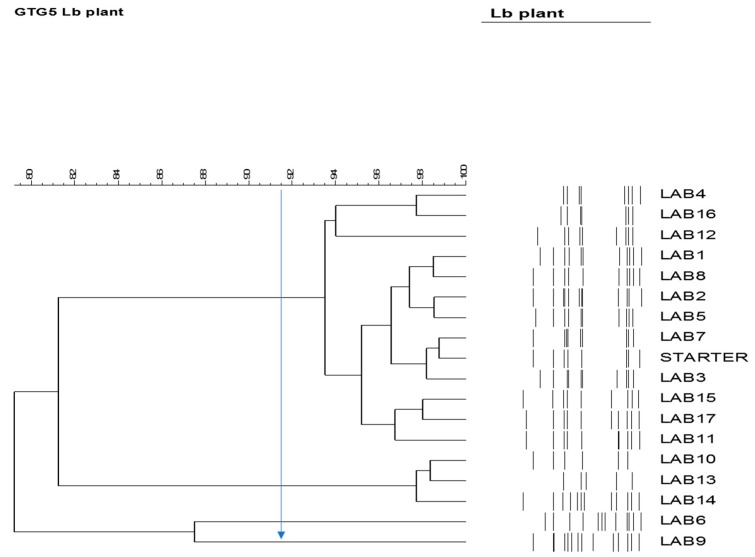
Dendrogram generated after cluster analysis of the digitized GTG5-PCR fingerprints of LAB (lactic acid bacteria) strains isolated from AL7 (LAB 6,9), AL8 (LAB 1,2,3,4,5,7,8), and AL9 (LAB 10,11,12,13,14,15,16,17) brine samples at 120 days of fermentation.

The presence of inoculated strain was monitored after 4 months of fermentation by rep-PCR on a pool of 17 strains from MRS agar (seven from AL8, eight from AL9, and two from AL7). Preliminarily, the repeatability of the method was confirmed using gDNA from the starter strain (Vegestart 60) as an internal control in four different gels from four different PCR reactions, obtaining a similarity of 91.8%. This value was retained as a threshold to establish the identity of isolates compared to the rep-PCR profile. The produced dendrogram clearly separated the studied strains into three clusters (Figure 10). More specifically, the first cluster related to the starter strain profile containing all isolates from AL9 (7/7, 100%) and many isolates from AL8 (5/8, 62.5%). The isolates of the second cluster belonged to AL8 (3/3), indicating that there were different strains, while isolates belonged to the third cluster came from AL7, in order to prove the distance between indigenous and starter LAB molecular profile.

## 4. Conclusions

According to the results of the present study, microbial, biochemical, and sensorial attitudes were strongly affected by brines inoculation, although a minor influence of salt content was also noted. The use of starter culture changed the microbial dominance and led to faster acidification of brines and faster degradation of oleuropein, indicating the faster fermentation completion. Moreover, the reduction of sodium content resulted in a successful lactic fermentation of Cypriot green cracked table olives. The final products fulfilled microbiological criteria and exhibited more appreciated sensorial characteristics. In addition, the formulation of table olives with low salt content is healthier and more suitable for consumers at risk of hypertension, opening a new era for table olives industry.

It must be mentioned that according to our findings, Cypriot olives were ready to eat after 120 days of fermentation in all treatments. This could be supported by the elimination of oleuropein, as well as the depletion of sugars (glucose and fructose), at this time point. Furthermore, the minor changes occurred after 120 days, confirming the above conclusion. Thus, the subsequent period was considered a preservation stage until selling, which was also important to be studied, in order to avoid any risk regarding the final product.

New methodologies used in olive and brine analysis (water potential and electrical conductivity) provided us with strong indications that they could be of interest as potential tools for the monitoring of the fermentation progress. However, further studies are required to establish a validated protocol.

Concluding, the effect of the inoculation of table olives on the production of stable quality and the final product was not dependent on the alternating indigenous microflora. The use of starter culture could lead to the modernization of fermentation, with healthier products of high quality. The study of the table olive indigenous microflora might lead to further research concerning its beneficial effects during olive processing with additional biotechnological and probiotic potential. Thus, further work is underway in order to study the multifunctional features of the isolated LAB and yeasts since indigenous microorganisms may be more adjusted to the harsh southeast Mediterranean environmental conditions while adding further to locally appreciated organoleptic characteristics.

## Figures and Tables

**Table 1:** Microbiological media used for microflora enumeration.

Growth Media	Microorganisms	Method	Incubation Conditions
Plate count agar (PCA) (Merck, Darmstadt, Germany)	Total viable count	Spread plate	30 °C/72 h
De Man-Rogosa-Sharpe agar (MRS) (Oxoid, Basingstoke, UK) + natamycin 0.1%	Lactic acid bacteria	Pour	30 °C/72 h
		plate/Overlay	
Sabouraud agar (Oxoid, Basingstoke, UK)	Yeast and Molds	Spread plate	25 °C/5 d
Violet red bile glycose agar (VRBGA)	*Enterobacteriacae*	Pour	37 °C/24 h
(BD, Sparks, MD)		plate/Overlay	
Violet red bile lactose agar (VRBLA)	*Coliforms*	Pour	30 °C/24 h
(Oxoid, Basingstoke, UK)		plate/Overlay	
Mannitol salt agar (MSA) (Oxoid, Basingstoke, UK)	*Staphylococci*	Spread plate	30 °C/48 h

## Supplementary Materials

The following are available online at https://www.mdpi.com/2304-8158/9/1/17/s1, Supplementary Table S1: Evolution of color parameters (a*, b*, L*, h*, and C*) of olive fruits during spontaneous (OL7), inoculated (10% NaCl) (OL8), and inoculated (7% NaCl) (OL9) fermentation of Cypriot green cracked table olives. Data points are expressed as means and standard deviations of 10 random measurements. Supplementary Table S2: Contribution of all studied variables to the factors in the PCA based on correlations.
