# Supplementary material for: Benefits of the Use of Lactic Acid Bacteria Starter in Green Cracked Cypriot Table Olives Fermentation
Source: Foods. 2019 Dec 23;9(1):17. doi: 10.3390/foods9010017 (PMC7023104; doi:10.3390/foods9010017)
Supplement: Supplementary file 1 [file foods-09-00017-s001.pdf]

Supplementary Table S1. Evolution of color parameters (a*, b*, L*, h* and C*) of olive fruits during spontaneous (OL7), inoculated (10% NaCl (OL8) and inoculated (7% NaCl) (OL9) fermentation of Cypriot green cracked table olives. Data points are expressed as means and standard deviations of 10 random measurements.

	0	8	15	22	29	45	60	90	120	150	210	281	365
OL7	-13,93±8,32	-7,58±1,11	-5,79±2,90	-3,37±0,92	-2,90±1,75	-1,29±1,04	-1,43±1,09	-1,02±1,43	-0,92±1,13	-1,90±0,99	-1,90±0,99	-1,80±0,92	-1,87±0,98
OL8	-13,5±2,31	-8,87±0,96	-8,24±1,58	-4,36±0,75	-3,50±0,41	-1,85±1,40	-1,23±0,88	-1,23±0,88	-0,92±0,50	-1,07±0,70	-1,40±0,38	-1,38±0,39	-1,40±0,38
OL9	-13,88±0,81	-7,51±0,76	-5,49±2,75	-4,37±0,04	-3,16±0,08	-1,11±1,07	-1,35±0,92	-1,35±0,92	-1,27±0,81	-1,69±0,46	-1,35±0,74	-1,35±0,62	-1,12±0,89
							**b***						
OL7	34,84±1,92	39,22±2,52	37,18±3,28	33,15±0,82	32,48±0,41	27,68±6,69	21,67±1,20	22,00±1,00	22,69±11,39	24,26±12,16	23,64±11,83	23,62±11,89	23,31±11,65
OL8	34,91±2,40	38,08±3,48	37,98±3,55	36,07±0,84	34,27±1,22	27,19±6,27	22,20±11,27	22,20±11,27	23,39±11,89	24,71±12,37	24,76±12,40	24,71±12,37	24,71±12,37
OL9	33,10±1,10	34,43±2,55	36,36±3,13	32,15±1,23	30,43±1,35	27,24±6,61	21,74±11,05	21,74±11,05	23,01±11,57	24,42±12,21	21,8±11,05	23,01±11,57	24,42±12,21
							**L***						
OL7	53,88±1,17	57,97±3,25	56,76±3,17	58,13±0,17	54,24±0,35	47,75±5,02	44,72±17,38	44,72±17,38	42,02±16,08	41,10±15,67	40,07±15,07	39,93±15,00	39,67±15,06
OL8	54,46±0,84	57,60±3,55	54,74±0,89	59,50±0,69	59,23±0,68	49,73±4,87	41,77±16,35	41,77±16,35	40,84±15,70	44,42±17,28	40,30±15,28	40,00±15,18	40,66±15,48
OL9	52,98±0,81	52,67±2,35	53,81±2,60	49,35±0,07	50,82±1,25	49,97±6,50	39,51±15,08	39,80±15,18	40,98±15,50	40,66±15,33	40,33±15,19	39,97±15,01	40,10±15,09
							**h***						
OL7	110,18±9,10	101,00±1,77	92,64±0,59	91,20±0,95	90,80±1,32	89,88±2,60	90,49±3,20	87,58±1,27	87,37±2,59	85,66±2,85	86,57±0,47	86,20±1,27	86,20±0,75
OL8	103,68±2,04	103,10±0,79	97,52±1,02	96,32±0,94	96,78±1,29	93,78±2,85	93,36±1,28	92,70±0,97	91,59±0,67	87,89±0,88	87,50±0,65	86,80±0,62	86,07±1,35
OL9	104,57±1,25	102,37±1,31	98,03±0,87	98,00±0,04	96,70±0,67	93,71±3,19	93,99±1,34	93,47±1,24	91,83±1,70	89,67±2,24	88,07±0,94	87,43±1,36	86,77±1,09
							**C***						
OL7	38,70±5,29	39,99±2,48	39,00±3,33	33,68±0,93	32,71±0,48	33,33±2,29	35,26±1,73	30,70±3,50	30,37±3,82	31,67±4,91	29,70±4,47	31,67±4,91	30,37±3,82
OL8	35,81±2,86	39,11±3,57	23,77±11,92	25,33±2,19	24,33±1,45	22,24±11,22	22,25±11,29	22,25±11,29	23,41±11,90	22,40±1,47	20± 2,3	19± 1,8	20±3,3
OL9	34,21±1,16	35,26±2,54	21,96±11,03	24,00±2,08	24,00±1,15	23,00±1,53	22,00±1,53	20,00±10,02	22,33±0,88	21,7±0,88	23,00±3,06	21,33±1,45	21,00±1,15

Supplementary Table S2. Contribution of all studied variables to the factors in the PCA based on correlations.

Component Matrix [a]				
	Component			
	1	2	3	4
LAB	-,791	-,493	,247	-,075
Yeasts	,514	,774	-,006	,168
Enterobacteriaceae	,793	-,318	-,427	-,054
pH	,256	,550	,642	-,390
Conductivity	-,245	-,474	-,188	,721
Water_potential	,335	-,463	-,104	-,535
Titration	-,696	-,667	-,041	-,060
Texture	,945	-,269	-,066	,024
Colour_a	-,847	,297	,212	,133
Colour_b	,896	-,218	,270	-,092
Colour_L	,743	-,155	,092	,001
Colour_h	,841	-,465	-,050	,134
Colour_C	,766	,374	-,323	,117
Total_polyphenols	,914	-,289	-,131	,109
Antioxidant_capacity	,836	-,202	,316	-,085
Oleuropein	,910	-,300	-,122	,126
Ydroxytyrosol	-,950	,101	,063	-,114
Lactic_acid	-,834	-,450	,050	,038
Acetic_acid	-,216	,916	-,230	-,042
Mallic_acid	,575	,325	,669	,137
Citric_acid	,895	,284	,056	-,005
Tartaric_acid	-,210	,541	,723	,138
Succinic_acid	-,574	-,628	,406	,039
Glucose	,663	,103	,678	,077
Fructose	,018	-,399	,806	,237
Ethanol	-,128	,956	-,176	,090
Glycerol	-,197	,932	-,183	,082

Extraction Method: Principal Component Analysis. a. 4 components extracted.
